# Extramitochondrial Assembly of Mitochondrial Targeting Signal Disrupted Mitochondrial Enzyme Aldehyde Dehydrogenase

**DOI:** 10.1038/s41598-018-24586-7

**Published:** 2018-04-18

**Authors:** Chalongrat Noree

**Affiliations:** Institute of Molecular Biosciences, Mahidol University, 25/25 Phuttamonthon 4 Road, Salaya, Phuttamonthon, Nakhon Pathom 73170 Thailand

## Abstract

Supramolecular assembly of metabolic enzymes has been studied both *in vivo* and *in vitro* for nearly a decade. Experimental evidence has suggested a close relationship between enzymatic activity and enzyme assembly/disassembly. However, most cases were studied with the cytosolic enzymes. Here, I report the evidence for a mitochondrial enzyme with its ability in forming visible intracellular structures. By removing the mitochondrial targeting sequence, yeast mitochondrial enzyme aldehyde dehydrogenase (Ald4p) exhibits reversible supramolecular assembly in the cytoplasm, thus creating a useful system for further characterization of the regulatory factors that modulate the assembly/disassembly of this mitochondrial enzyme.

## Introduction

Several pieces of evidence have shown that many enzymes possess multi-function characteristics. Other than their well-characterized biochemical property, they might serve in other unrelated biological processes, and they have been grouped as “moonlighting enzymes”^1–3^. The first described moonlighting enzyme was lactate dehydrogenase (LDH), performing its non-canonical function as structural protein in the epsilon-crystalline, found in duck lens^4^. For another example, pyruvate kinase (PK), typically catalyzing pyruvate production in the cytoplasm, can be translocated into the nucleus, moonlighting the epigenetic function in regulation of specific gene expression^5^. Not only pyruvate kinase, other glycolytic enzymes have previously been reported about their presence in the nucleus with newly discovered roles, unrelated to their typical glycolytic function^6^. These cumulative findings have suggested the unprecedented potential of metabolic enzymes to be adaptively versatile in response to environmental changes.

Intracellular structures formed by cytosolic enzymes have been continuously reported in several organisms, for examples, CTP synthetase filaments identified in bacterial cells^7^, asparagine synthetase (Asn2p), glutamine synthetase (Gln1p), phosphoribosylpyrophosphate amidotransferase (Ade4p) puncta^8^, CTP synthetase (Ura7p/Ura8p), glutamate synthase (Glt1p), GDP-mannose pyrophosphorylase (Psa1p) filaments^9^, glutamine synthetase (Gln1p) filaments^10^, and asparagine synthetase (Asn1p/Asn2p) filaments^11^ in yeast cells, CTP synthetase filaments in flies^9,12^, CTP synthetase filaments in rat neuronal cells^9^, CTP synthetase (CTPS1) with inosine monophosphate dehydrogenase (IMPDH2) rods and rings^13^, and co-clusters of *de novo* purine biosynthetic enzymes in human cell lines^14^. These discoveries have suggested the unknowingly extra roles of these enzymes in addition to their well-characterized biochemical functions. Furthermore, their assembly/disassembly might be associated tightly with the regulations of their enzymatic activity.

During my latest re-screen of the yeast GFP collection, constructed by O’Shea and Weissman^15^, several metabolic enzymes have been found that they could exhibit the supramolecular assembly (unpublished data). Surprisingly, not only cytosolic enzymes were microscopically visualized showing their visible intracellular structures, but might also be the case for some mitochondrial enzymes. Since the mitochondria are space-limited, I came up with an idea to inhibit the selected yeast mitochondrial enzyme, aldehyde dehydrogenase Ald4p, from being targeted to its residential compartment such that it can occupy a larger space, in cytoplasm, to exhibit supramolecular assembly.

Aldehyde dehydrogenase is a key enzyme responsible for metabolizing a toxic and stress inducible substance, acetaldehyde, produced during yeast fermentation by converting it to acetate. There are several isoenzymes of yeast aldehyde dehydrogenase and they can be categorized based on their subcellular locations. Cytosolic aldehyde dehydrogenases are encoded by *ALD2*, *ALD3*, and *ALD6*, whereas the mitochondrial counterparts are encoded by *ALD4* and *ALD5*^16^. *ALD4* and *ALD6* are two major contributors, however, their expressions are distinctly activated. *ALD6* expression is activated when glucose is used as a carbon source. In contrast, the availability of glucose leads to repression of *ALD4* expression. Expression of *ALD4* can be induced in the presence of ethanol and acetaldehyde^17^.

Here, I provide another piece of evidence supporting the existence of extramitochondrial assembly of MTS-deleted Ald4p-GFP. Furthermore, this could be a system for further investigations of the regulatory factors promoting or inhibiting the assembly of any mitochondrial enzyme being studied.

## Results and Discussion

### Sporadic distribution of aldehyde dehydrogenase (Ald4p) could be observed in yeast mitochondria

From re-screening the yeast GFP collection to search for any GFP-tagged metabolic enzyme capable of supramolecular assembly, I found that yeast mitochondrial enzyme aldehyde dehydrogenase (Ald4p), involved in acetate biosynthesis, and also, ethanol and pyruvate metabolism^16^, seemed likely to possess this property. Through the literature search, this enzyme has recently been reported that it is a component of the needle-like structures, present in fused mitochondria^18^.

However, limited by small space of the mitochondria, it is difficult to test whether Ald4p can actually assemble into long filaments if the enzyme is still being targeted to its natural compartment. By removing the MTS from its coding sequence in the yeast genome, the produced enzyme cannot be targeted to the mitochondria. Simultaneously, by introducing GFP after the gene, this allows visualization of its assembly under the fluorescence microscope. However, GFP is a fluorescent tag with a concern that it might cause the artefactual assembly of any GFP-tagged protein when overexpressed. But, in my case, I designed to express GFP fusion protein using PCR-based engineering of yeast genome^19^. This technique (Supplementary Fig. S1) enables the enzyme, still, to be made from its original single copy under the control of its endogenous promoter and also ensures that the structure formation of MTS-deleted Ald4p-GFP [from now on, dubbed “Ald4p(noMTS)-GFP”] would not be a result of overexpression. As expected, the levels of cytoplasmically targeted Ald4p(noMTS)-GFP, in the yeast *ALD4(noMTS)::GFP*, were not overly expressed, and actually, showed much less than those of a typical mitochondrial Ald4p-GFP, in the yeast *ALD4::GFP* (Supplementary Fig. S2).

In addition, some other Ald4p counterparts, Ald2p-GFP and Ald3p-GFP (the amino acid sequences of Ald2p and Ald3p both share about 60% similarity with that of Ald4p, Supplementary Fig. S3) do not assemble into high-order structures in any stages of growth (unpublished data). Altogether, these argue that the supramolecular assembly of Ald4p(noMTS)-GFP is not driven by GFP.

### MTS-deleted Ald4p-GFP exhibited high-order structure formation in the yeast cytoplasm

**Figure 1:**
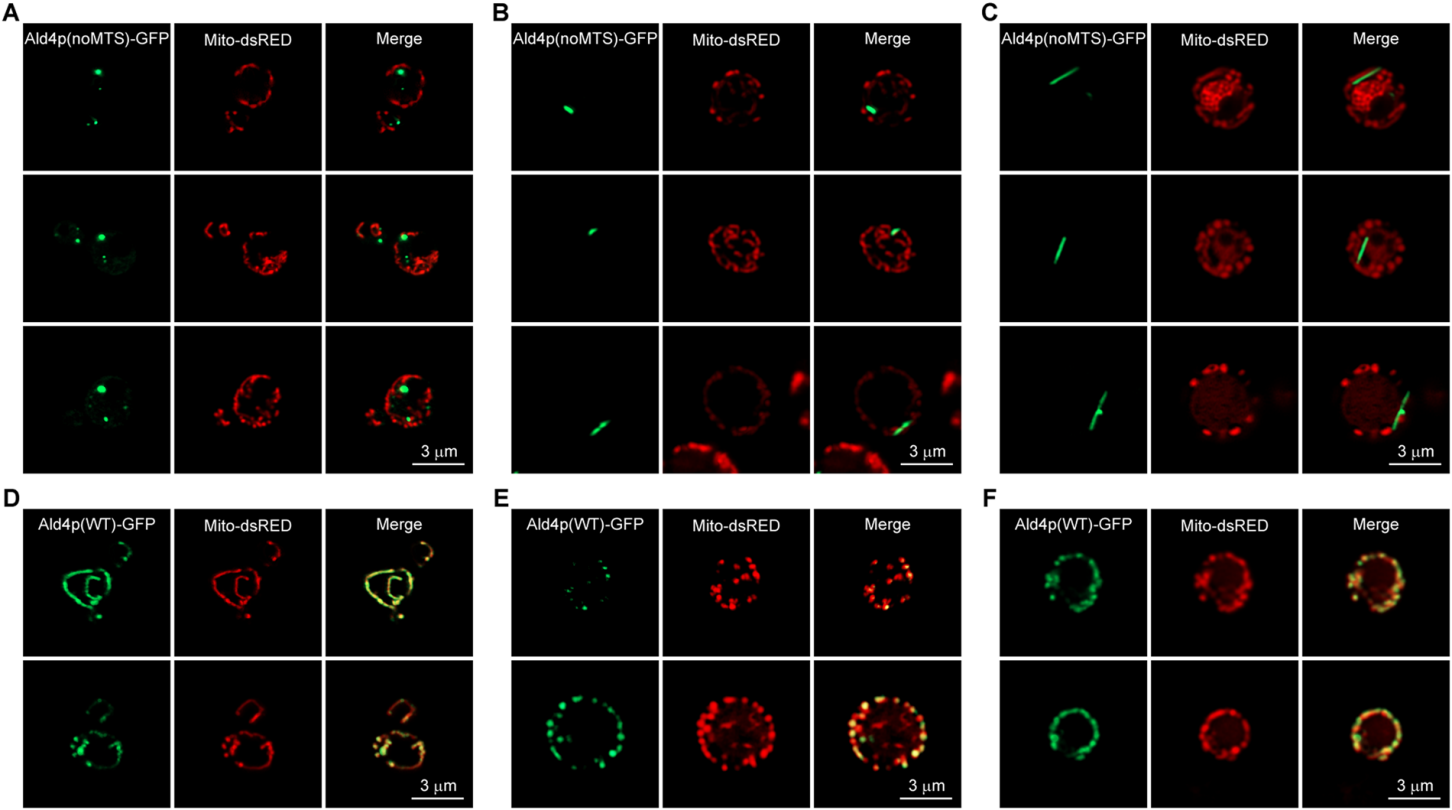
Live cell images showing cytoplasmic puncta, rods, and filaments of Ald4p(noMTS)-GFP observed in yeast *ALD4(noMTS)::GFP* grown in SC-uracil medium for 1 day, 3 days, and 7 days, respectively. Yeast *ALD4(noMTS)::GFP* transformed with pVTU-mito-dsRED (**A**–**C**) and *ALD4(WT)::GFP* transformed with pVTU-mito-dsRED, as a control (**D**–**F**) were grown in liquid uracil-dropout medium at 30 °C for 1 day (**A**,**D**), 3 days (**B**,**E**), and 7 days (**C**,**F**) with shaking, captured live in Z-stack for approximately 1–3 μm, and projected into 2D image using maximum intensity.

After verifying the permanent removal of MTS from the chromosomal *ALD4* by PCR and DNA sequencing (Supplementary Fig. S4), the yeast construct *ALD4(noMTS)::GFP* was transformed with a plasmid expressing dsRED in the mitochondria (pVTU-mito-dsRED). This was another confirmation on the success of relocation of the Ald4p(noMTS)-GFP from the mitochondria to the cytoplasm. Ald4p(noMTS)-GFP showed no colocalization pattern with the mitochondria, as it did for the typical Ald4p-GFP (Fig. 1).

In observations throughout their growth stages, visible cytoplasmic puncta of Ald4p(noMTS)-GFP could be seen, but in less than 10% of log-phase cell population. Once the cells were continually grown to saturation, most of the cells (>90%) carried Ald4p(noMTS)-GFP structures (0.35 μm on average, 1-day culture) (Fig. 1A), and some started to have giant rod-like structures (0.6–1.4 μm, 3 days onward) present in the cytoplasm (Fig. 1B). Interestingly, long filaments (1.5–3.9 μm) could later be found clearly after growing the cells for 3–7 days (Fig. 1C). This reorganization of Ald4p(noMTS)-GFP by transitioning from puncta to rod-shaped structures and finally to long filaments is very intriguing for further investigations. Would these different shapes be reflecting different regulatory states and functions of the enzyme?

### Ald4p(noMTS)-GFP structures dynamically responded to environmental changes

**Figure 2:**
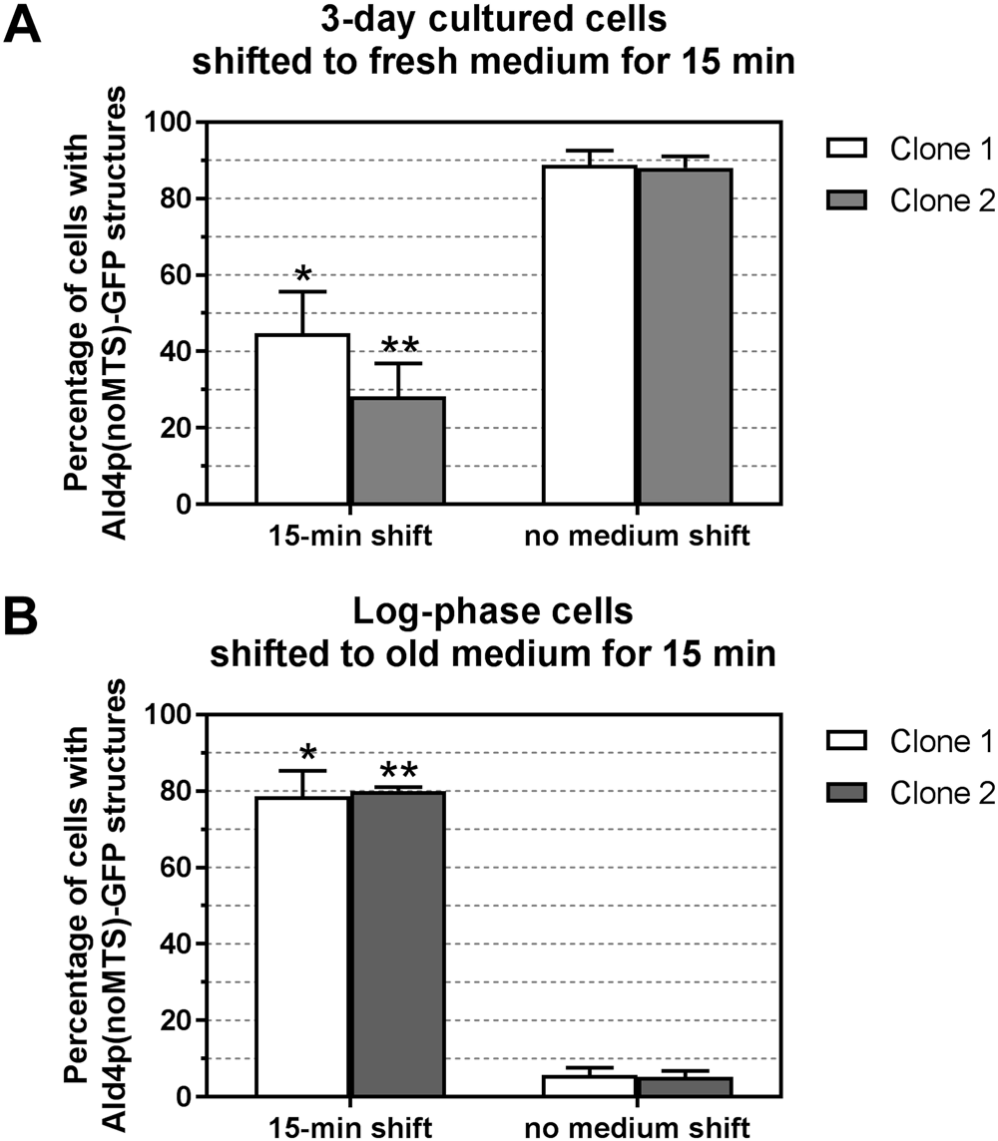
Ald4p(noMTS)-GFP disassembled after the cells were shifted to fresh medium, but assembled more after shifted to old medium. (**A**) Yeast *ALD4(noMTS)::GFP* transformed with pVTU-mito-dsRED was grown in liquid uracil-dropout medium at 30 °C for 3 days with shaking. The cells were then shifted to fresh medium and incubated at 30 °C for 15 min with shaking before counting. (**B**) Yeast *ALD4(noMTS)::GFP* transformed with pVTU-mito-dsRED was grown in liquid uracil-dropout medium to log-phase stage of growth at 30 °C with shaking. The cells were then shifted to old medium and incubated at 30 °C for 15 min with shaking before counting. The cells were inspected under fluorescence microscope for percentage of cells with Ald4p(noMTS)-GFP structures, comparing between 15-min shifted and non-shifted conditions. Two different clones were used in the experiments. For each clone, three independent experiments were performed and reported as average ± SEM (Raw data and statistical analyses shown in Supplementary Table S2).

To investigate whether this mitochondrial enzyme behaves similarly to other cytoplasmic enzymes previously reported about their reversible assembly^8–10,14^, the yeast *ALD4(noMTS)::GFP* with pVTU-mito-dsRED was first grown for 3 days in SC-uracil, then switched into a fresh medium for 15 min. As expected, the cells shifted to the fresh medium showed the number of cells with Ald4p(noMTS)-GFP structures about a half of those without medium change (Fig. 2A).

Conversely, the log-phase *ALD4(noMTS)*::*GFP* cells shifted to the old medium, isolated from 3-day-old cultures, for 15 min were obviously observed for the 13-fold increase in the number of cells with Ald4p(noMTS)-GFP structures, when compared to the control, cells without medium change (Fig. 2B). These results indicated that the structures formed by Ald4p(noMTS)-GFP were not just perpetual inclusion bodies.

It is interesting to note that the SC-uracil medium isolated from 1-, 3-, and 7-day cultures of yeast *ALD4(noMTS)::GFP* demonstrated a progressive drop in their pH values (from pH of around 5 to 3), compared with the control, fresh SC-uracil (pH being in a range of 5–6) (Supplementary Fig. S5). This is in agreement with the previous finding that acidic pH promotes the assembly of metabolic enzymes into cytoplasmic filaments, whereas neutral/basic pH contributes to their disassembly, suggesting the transitional adaptations of cells to survive during starvations and to re-enter the cell cycle upon nutrient-rich conditions via metabolic reorganization in their cytoplasm^10^.

### Extramitochondrial relocation of mitochondrial enzymes can serve as an alternative for increasing productivity of the desired metabolic products and also for characterizing their supramolecular assembly regulatory factors

Not only Ald4p, the mitochondrial enzyme acetolactate synthase (Ilv2p), has also been reported about its ability to form visible structures in the cytoplasm, following the metabolic engineering attempts to relocalize Ilv2p and also the enzymes located downstream of the pathway to the cytoplasm so that the rate-limiting transportation step of their pathway intermediates across the mitochondrial membranes can be bypassed, thus leading to a significant increase in alcohol productivity^20^.

Another mitochondrial enzyme, E1 alpha subunit of the pyruvate dehydrogenase complex (Pda1p), was a hit from a high content screen of the progenies derived from crossing the selected yeast GFP collection with the synthetic genetic array (SGA) yeast that showed spatially organized as visible puncta in the mitochondrial matrix, and being in close proximity to peroxisomes. This has implicated the metabolic function coordination between these two compartments^21^ in energy production with the breakdown of fatty acids, in the peroxisome, into acetyl-coA and the TCA cycle, in the mitochondria.

In consistent with the findings above, the evidence for mitochondrial enzymes to assemble into supramolecular structures has been cumulatively added here, thus supporting the common feature of this cellular process possessed by both cytoplasmic and mitochondrial enzymes. Also, the relocation of mitochondrial enzyme to be in the cytoplasm has established a feasible system that can be used for further investigations of the regulatory factors involved in assembly and disassembly events. And this might shed light onto the modulation of enzymatic activity through the enzyme assembly.

## Methods

### Bacteria, yeast strain, growth and selection media

*Escherichia coli* DH5α was used for cloning the recombinant plasmids. LB medium [0.5% (w/v) yeast extract (BD), 1% (w/v) Bacto-tryptone (BD), 1% (w/v) sodium chloride (BDH Prolabo)] supplemented with 100 μg/ml ampicillin (PanReac Applichem) was used for selection. Bacterial cultures were maintained at 37 °C.

Yeast BY4741 (MATa his3Δ1 leu2Δ0 met15Δ0 ura3Δ0), used as a background strain for yeast chromosomal gene modifications. YPD medium [1% (w/v) yeast extract (BD), 2% (w/v) Bacto-peptone (BD), and 2% (w/v) dextrose (Sigma-Aldrich)] was used for general growth. G418 (PanReac Applichem) was used for selecting yeast transformants. Uracil-dropout medium (Sigma-Aldrich) “SC-uracil” containing 2% (w/v) dextrose was used to select for yeast transformed with pVTU-mito-dsRED. All yeast constructs were grown at 30 °C.

### Construction of recombinant plasmid

pFA6a-GFP-kanMX6 (a gift from J. Wilhelm, UCSD) was used for molecular cloning of *ALD4*. The *ALD4* coding sequence was amplified by PCR from the isolated genomic DNA of yeast BY4741, using KOD Hot Start DNA Polymerase (Merck), and then directionally cloned into pFA6a-GFP-kanMX6 at *Sal*I and *Sma*I restriction recognition sites. The resulting plasmid was named pFA6a-ALD4-GFP-kanMX6. Primers (Macrogen, South Korea) used for PCR are shown in Supplementary Table S1.

### Yeast chromosomal gene modification

The information of nucleotide sequence coding for MTS was retrieved from the UniProt database (http://www.uniprot.org/). MTS of *ALD4* is encoded by nt1-72.

PCR-based engineering of the yeast genome^19^ was employed to construct the yeast *ALD4(no MTS)::GFP*. First, pFA6a-ALD4-GFP-kanMX6 was used as a DNA template for making the DNA cassette harboring (sequence in order from 5′ to 3′): 50 nt upstream of the *ALD4* start codon, ATG, *ALD4* coding sequence (without MTS), GFP, kanamycin resistance gene, and 50 nt downstream of the *ALD4* stop codon. PCR reaction was set up using the KOD Hot Start DNA Polymerase kit (Merck). Yeast BY4741 was transformed with the purified DNA cassette using lithium acetate/PEG transformation method^22^. YPD with 400 μg/ml G418 was used for selection. Positive (MTS-deleted) and negative (intact MTS) yeast transformants were initially screened under the fluorescence microscope by checking two distinct localization patterns (cytosolic and mitochondrial, respectively), and were then confirmed by sending out the PCR product of the isolated genomic DNA from the yeast construct to be verified for DNA sequencing (Macrogen, South Korea) (Supplementary Fig. S3). Primers for making the DNA cassette for yeast transformation and sequencing primers are shown in Supplementary Table S1.

### Western blot analysis

To ensure that the extramitochondrial assembly of Ald4p(noMTS)-GFP was not caused by overexpression, *ALD4(noMTS)::GFP* and *ALD4(WT)::GFP*, 3 different clones for each strain, were cultured in YPD at 30 °C for 1 day with shaking. Five OD_600_ cells were taken from each culture for preparing whole cell extract. After centrifugation at 6,000 rpm for 3 min and removal of supernatant, cells were lysed in 200 μl SDS-PAGE sample buffer containing (1:20) beta-mercaptoethanol (PanReac Applichem) and (1:1000) protease inhibitor cocktail (Sigma-Aldrich). About 50 μl glass beads (425–600 μm) (Sigma-Aldrich) were added to help in cell lysis during the vigorous vortex for 1 min. After boiling protein samples at 95 °C for 10 min and placing on ice for 5 min, they were centrifuged at 10,000 rpm for 1 min at room temperature. Five μl of each protein sample was resolved on 8% SDS-PAGE. AccuProtein Chroma prestained protein marker (Enzmart Biotech) was used to identify the approximate size of Ald4p(noMTS)-GFP, Ald4p(WT)-GFP, and alpha tubulin. The resolved proteins were transferred to PVDF membrane (Bio-Rad) using ECL Semi-Dry Transfer Unit TE70 (Amersham Biosciences). Western blotting was performed using standard protocol. Rabbit anti-GFP polyclonal antibody (A01388; GenScript; 1:5,000) was used to detect GFP-tagged Ald4p(noMTS) and Ald4p(WT). Mouse anti-alpha tubulin (12G10; DSHB, University of Iowa; 1:5,000) was used for showing internal loading control. The secondary antibodies for detection were HRP-conjugated goat anti-rabbit IgG (Sigma-Aldrich; 1:5,000) and HRP-conjugated goat anti-mouse IgG (Sigma-Aldrich; 1:5,000), respectively. ECL Start Western Blotting Detection System (GE Healthcare) was used to develop the chemiluminescent signals before exposure to X-ray films (Fujifilm). Three independent experiments were performed to confirm the results.

### Colocalization assay

To confirm the successful inhibition of MTS-deleted and GFP-tagged Ald4p from getting into its residential compartment “mitochondria”, yeast *ALD4(noMTS)::GFP* was transformed with a mitochondrial marker plasmid “pVTU-mito-dsRED” (a gift from J. Wilhelm, UCSD) using lithium acetate/PEG transformation method (500 ng plasmid for each transformation). SC-uracil with 400 μg/ml G418 was used for selecting yeast transformants.

### Media shift assay

To study disassembly of Ald4p(noMTS)-GFP, yeast *ALD4(noMTS)::GFP* transformed with pVTU-mito-dsRED was grown in SC-uracil at 30 °C for 3 days with shaking at 250 rpm (Green SSeriker II Model VS-8480FSN, Vision Scientific). An aliquot of 3-day culture was transferred to a microfuge tube, centrifuged at 6,000 rpm for 2 min at room temperature (Centrifuge 5424, Eppendorf). After discarding the old medium, cells were resuspended in the same volume of fresh SC-uracil. The cells were incubated at 30 °C for 15 min with shaking at 300 rpm (ThermoMixer C, Eppendorf). The control was processed similarly, except that there was no step of medium shift.

For Ald4p(noMTS)-GFP assembly induction assay, yeast *ALD4(noMTS)::GFP* transformed with pVTU-mito-dsRED was cultured in SC-uracil to log-phase stage of growth at 30 °C with shaking. An aliquot of log-phase culture was transferred to a microfuge tube, centrifuged at 6,000 rpm for 2 min at room temperature. After discarding the old medium, cells were resuspended in the same volume of 3-day-old SC-uracil. The cells were incubated at 30 °C for 15 min with shaking. Noting that the 3-day-old medium, used in this assay, was collected from the 3-day culture. Briefly, the 3-day culture was centrifuged at 10,000 rpm at room temperature for 3 min. Only the clear liquid medium was carefully transferred to a sterile microcentrifuge tube, followed by centrifugation again at 10,000 rpm for 3 min at room temperature. The clear liquid medium from the second centrifugation was carefully transferred to a new sterile microcentrifuge tube and used to treat the cells immediately. The control was processed similarly, except that the step of medium shift was skipped.

About 10–15 μl of cells were put on a microscope slide (Shandon Superfrost Plus, Thermo Scientific), covered by a coverslip (Menzel Gläser, Thermo Scientific), and the excess liquid was blotted off before analysis under fluorescence microscopy. Cell counting method, data presentation, and statistical analyses were performed as described previously^23^. Briefly, the cells were randomly counted in 5 different fields, and about 50 cells per field (about 250 cells in total) were counted in each experiment. Then, the percentage of cells with Ald4p-GFP structures was calculated. The average ± SEM of three independent experiments was reported for each condition, and student’s t-test was used for statistical analyses.

### Monitoring pH values of media isolated from 1-, 3-, and 7-day yeast cultures

Yeast *ALD4(noMTS)::GFP* transformed with pVTU-mito-dsRED was grown in SC-uracil at 30 °C for 1, 3, and 7 days with shaking at 250 rpm. Aliquots of 1-, 3-, and 7-day cultures were transferred to sterile microcentrifuge tubes, centrifuged at 10,000 rpm for 3 min at room temperature. Only the clear liquid medium from each culture was carefully transferred to a new sterile microcentrifuge tube, followed by centrifugation again at 10,000 rpm for 3 min at room temperature. The clear liquid medium from the second centrifugation was carefully transferred to a new sterile microcentrifuge tube and used to check its pH value immediately with pH-indicator strips (Merck). Three independent experiments were performed to confirm the results.

### Cell imaging

Yeast cells were grown to the growth stages or experimented as indicated. Wet slides were prepared by dropping the live cell suspension onto a slide, covering with a coverslip, blotting off excess liquid to prevent cells from floating around, and then sealing edges of the coverslip with nail polish. Images were taken with the Carl Zeiss LSM800 with AiryScan using Plan-Apochromat 63 × /1.4 Oil DIC ∞/0.17 objective lens with Zen Blue software version 2.1.57.1000. Quantification of Ald4p(noMTS)-GFP structures was performed as described previously^23^.

### Data Availability Statement

All data generated or analyzed during this study are available from the corresponding author on reasonable request.

## Electronic supplementary material

Supplementary Figures and Tables
