# Supplementary material for: Extramitochondrial Assembly of Mitochondrial Targeting Signal Disrupted Mitochondrial Enzyme Aldehyde Dehydrogenase
Source: Sci Rep. 2018 Apr 18;8:6186. doi: 10.1038/s41598-018-24586-7 (PMC5906672; doi:10.1038/s41598-018-24586-7)
Supplement: Supplementary file 1 — Supplementary Figures and Tables [file 41598_2018_24586_MOESM1_ESM.docx]

**Supplementary Information**

**Extramitochondrial Assembly of Mitochondrial Targeting Signal Disrupted Mitochondrial Enzyme Aldehyde Dehydrogenase**

Chalongrat Noree*

Institute of Molecular Biosciences, Mahidol University, 25/25 Phuttamonthon 4 Road, Salaya, Phuttamonthon, Nakhon Pathom, 73170 Thailand

*Correspondence:

Chalongrat Noree

Tel: +66-2-4419003

Fax: +66-2-4411013

Email: chalongrat.nor@mahidol.edu

**
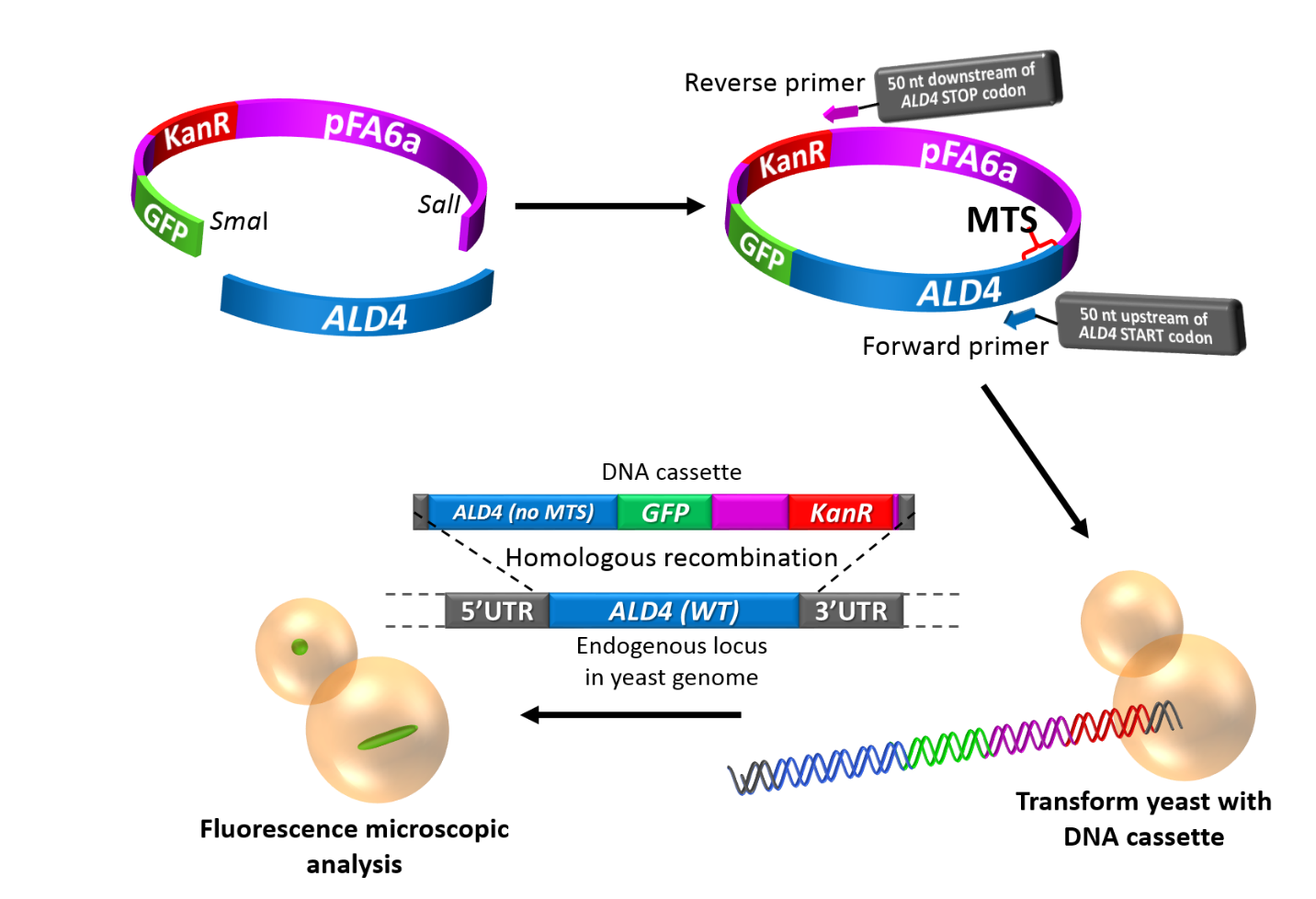
**

**Supplementary Figure S1. Experimental Design.** PCR-based modification of the yeast genome was used to delete the MTS coding sequence from chromosomal *ALD4*, and simultaneously introduce GFP to 3’ of the gene for visualization under fluorescence microscope. MTS-deleted Ald4p-GFP was then observed whether they could form supramolecular structures in the cytoplasm.

**A**

**
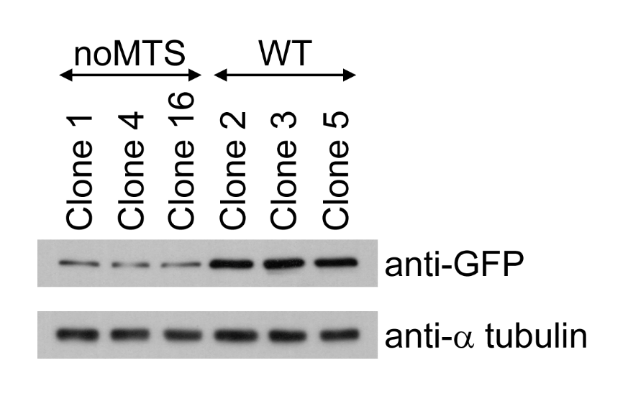
**

**
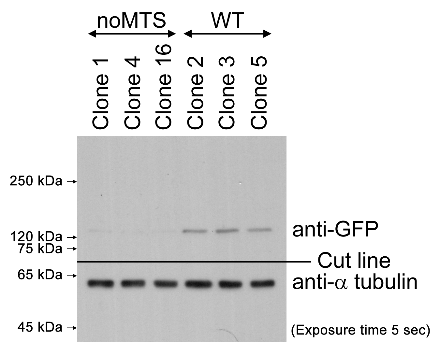

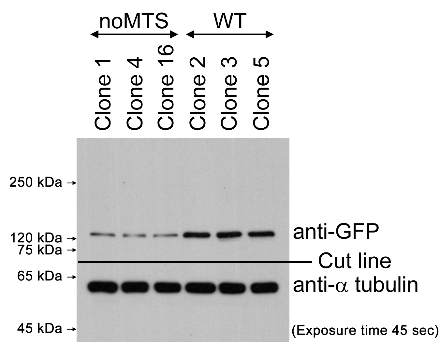

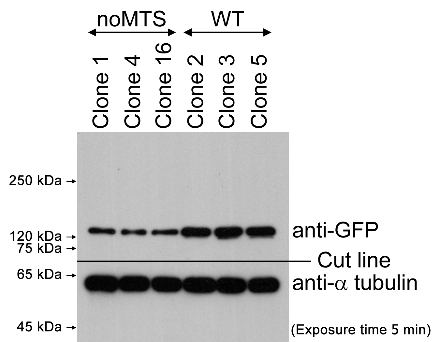
**

**B**


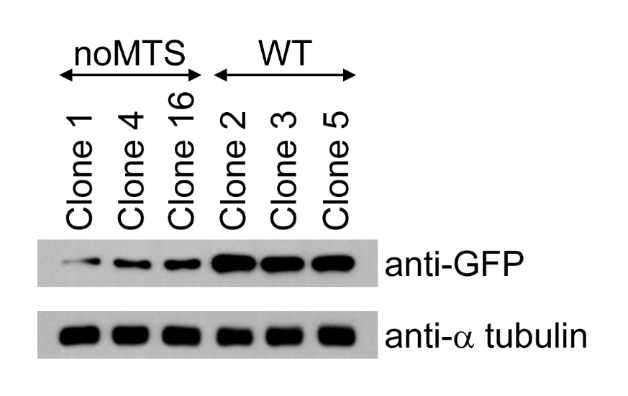


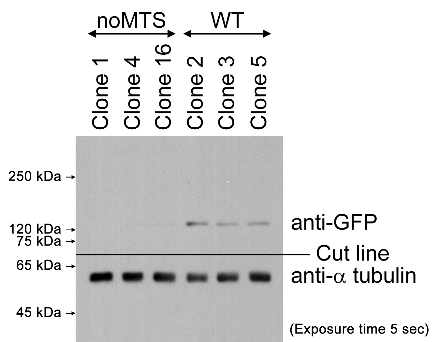

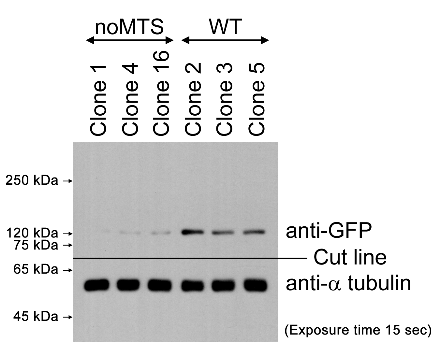

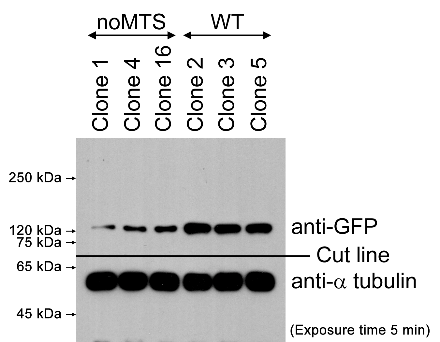


**C**


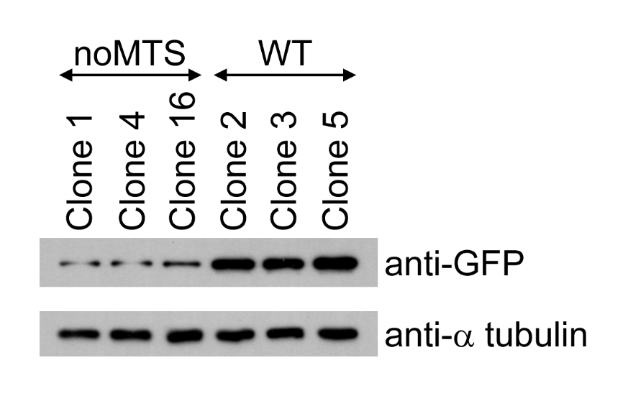


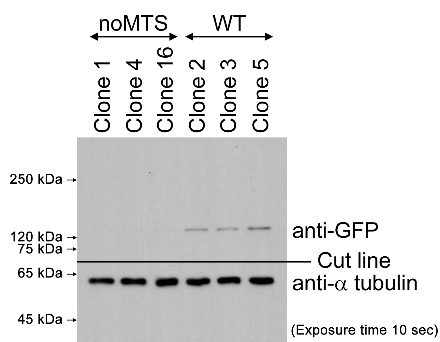

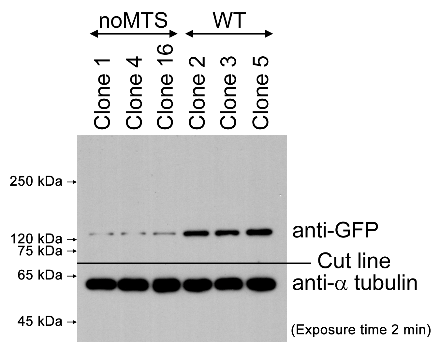

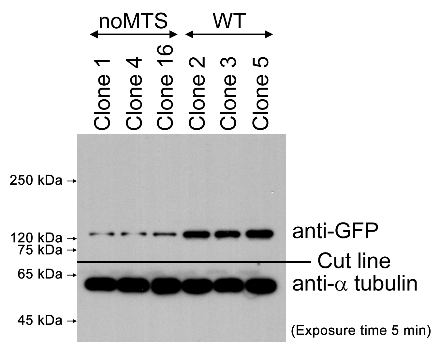


**Supplementary Figure S2.** **Extramitochondrial structure formation of Ald4p(noMTS)-GFP was not driven by overexpression.** Three different clones of yeast *ALD4(noMTS)::GFP* and *ALD4(WT)::GFP* were grown in liquid YPD at 30°C for 1 day with shaking. Then, 5 OD_600_ cells were taken from each culture to prepare whole cell extract for SDS-PAGE and Western blot analysis. Each membrane was cut into 2 pieces between 75 and 65 kDa of the pre-stained protein ladder. The upper cuts were used to detect GFP-tagged Ald4p(noMTS) and Ald4p(WT) with anti-GFP (81.4 kDa and 84.0 kDa, respectively). The bands of Ald4p(noMTS)-GFP and Ald4p(WT)-GFP were detected slightly above the 120-kDa band of the protein ladder, suggesting that they were being in a dimeric form. The lower cuts were used to detect alpha-tubulin (as internal loading control) (49.7 kDa). Both were later assembled and developed the chemiluminescent signal together. (**A**), (**B**), and (**C**) were three different blots from three independent experiments performed to confirm the results. Full blots with multiple exposure times are shown below the cropped blots.

**
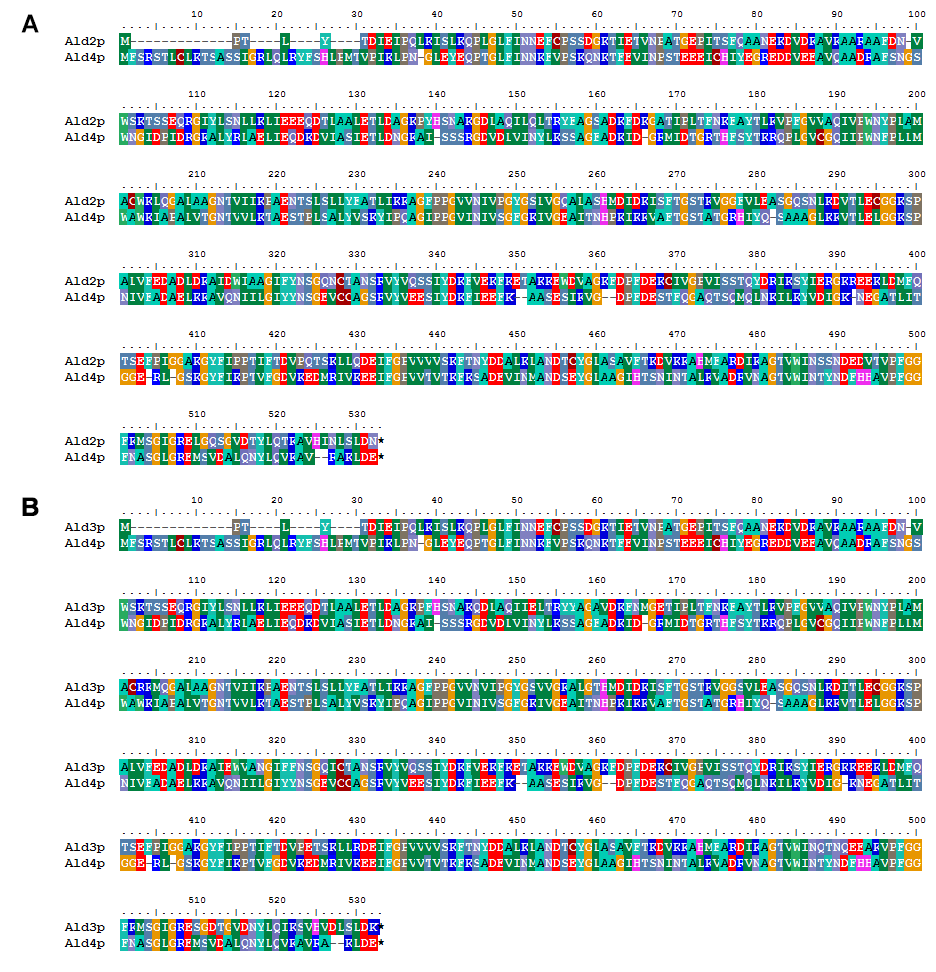
**

**Supplementary Figure S3.** **Pairwise amino acid sequence alignments of aldehyde dehydrogenase isoenzymes.** (**A**) The alignment of Ald2p (YMR170C, SGDID:S000004780) and Ald4p (YOR374W, SGDID:S000005901) shows 43.7% identity and 59.7% similarity in their amino acid sequences. (**B**) The alignment of Ald3p (YMR169C, SGDID:S000004779) and Ald4p (YOR374W, SGDID:S000005901) shows 42.4% identity and 59.3% similarity in their amino acid sequences.

**
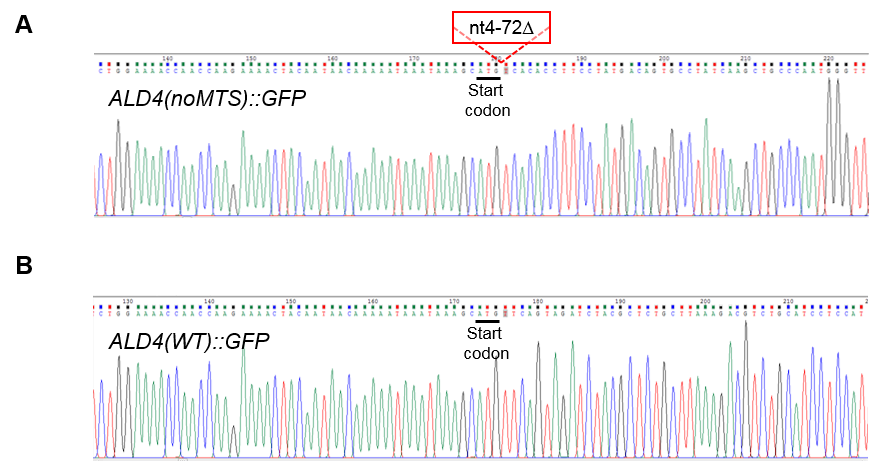
**

**Supplementary Figure S4.** **Verification of mitochondrial targeting signal removed from the chromosomal gene *ALD4* by DNA sequencing.** (**A**) The nucleotides 4 to 72 of *ALD4* coding sequence successfully deleted. (**B**) The wild-type sequence of *ALD4* (as control).

**
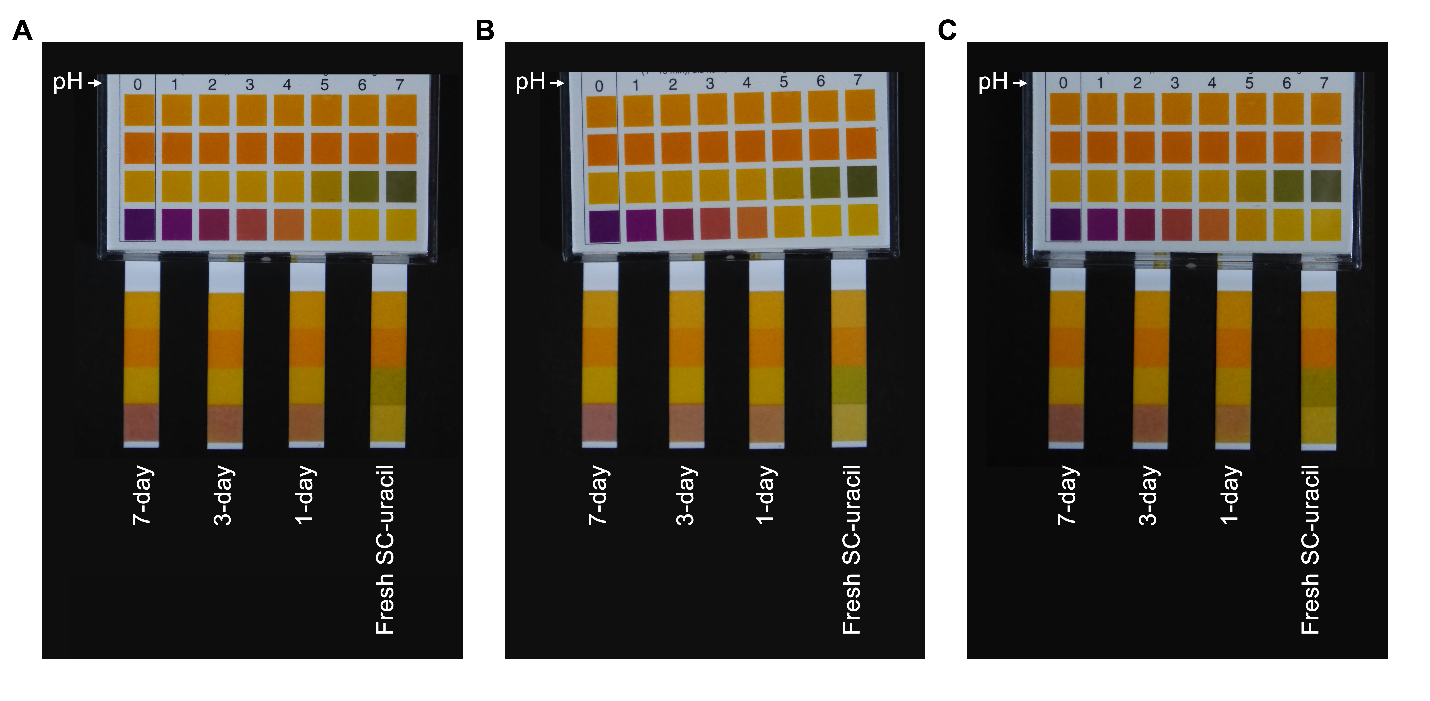
**

**Supplementary Figure S5.** **Progressive drop of pH values of yeast culture medium SC-uracil.** Yeast *ALD4(noMTS)::GFP* transformed with pVTU-mito-dsRED was grown in SC-uracil at 30°C with shaking for 1 day, 3 days, and 7 days, respectively. The culture media at different time points were isolated and tested for their pH values with pH 0-14 universal indicator strips. (**A**), (**B**), and (**C**) were three independent experiments.

**Supplementary Table S1.** **List of primers used for recombinant DNA cloning, making DNA cassette for yeast transformation, and DNA sequencing.**

| **Primer Code** | **Sequence (5’ to 3’)** | **Description** | **Used with** | **PCR Product Size** |
| --- | --- | --- | --- | --- |
| **For cloning *ALD4* into pFA6a-GFP-kanMX6 (resulting plasmid: pFA6a-ALD4-GFP-kanMX6)** | | | | |
| CN0001 | 5’- GGGGTCGACATGTTCAGTAGATCTACGCTCTG -3’ | Forward, for cloning ***ALD4***, ***Sal*I** site underlined. | CN0002 | 1,575 bp |
| CN0002 | 5’-AAACCCGGGCTCGTCCAATTTGGCACGG-3’ | Reverse, for cloning ***ALD4***, ***Sma*I** site underlined. | CN0001 |  |
| **For making DNA cassette carrying (5’ to 3’): 50 nt upstream of *ALD4* start codon + *ALD4* coding sequence (*without nucleotides coding for MTS*) + GFP + kanamycin resistance gene + 50 nt downstream of *ALD4* stop codon**  **DNA template: pFA6a-ALD4-GFP-kanMX6** | | | | |
| CN0003 | 5’- GTATCTGGAAAACCAACCAAGAAAACTACAATAACAAAAATAAATAAAGC**ATG**TCACACCTTCCTATGACAGTG -3’ | Forward, with 50 nt upstream of ***ALD4*** start codon, **nt73** (underlined) is placed right after start codon. | CN0004 | 4,012 bp |
| CN0004 | 5’- TTAATTTTATGTATGTAAGCATCGATTGGACACCAGGCTTATTGATGACCATCGATGAATTCGAGCTCG -3’ | Reverse, with 50 nt downstream of ***ALD4*** stop codon, sequence homology to pFA6a-ALD4-GFP-kanMX6 (underlined). | CN0003 |  |
| **For PCR verification of yeast transformants (to check if MTS is removed from *ALD4*, in the yeast genome)** | | | | |
| CN0009 | 5’- GGAAAATGAGGGGCGGGTGTAG -3' | Forward; 200 nt upstream of ***ALD4*** start codon. | JW1623 | 2670 bp (having MTS), 2601 bp (MTS removed) |
| JW1623 | 5’- GCGACCTCATACTATACCTG -3’ | Reverse; 164 nt downstream of **GFP** stop codon. | CN0009 |  |
| **For DNA sequencing** | | | | |
| CN0009 | 5’- GGAAAATGAGGGGCGGGTGTAG -3' | Forward; 200 nt upstream of ***ALD4*** start codon | | |
| CN0010 | 5’- AAGATTGCCCCTGCTTTGG -3’ | Forward; nt 601-619 of ***ALD4*** coding sequence | | |
| CN0011 | 5’- TACTTCATTAAGCCAACTGTCTTTG -3’ | Forward; nt 1201-1225 of ***ALD4*** coding sequence | | |

**Supplementary Table S2. Data, used to plot graphs in Fig. 4, showing percentage of yeast cells *ALD4(noMTS)::GFP* with Ald4p(noMTS)-GFP structures comparing between media-shifted and non-shifted conditions.**

**(A)** **Three-day cultured cells shifted to fresh medium for 15 min.**

| **Experiment #** | **Clone 1** | | **Clone 2** | |
| --- | --- | --- | --- | --- |
|  | **Shifted to fresh medium** | **No medium shift** | **Shifted to fresh medium** | **No medium shift** |
| 1 | 23.11% | 96.21% | 29.02% | 93.80% |
| 2 | 58.47% | 84.27% | 12.89% | 86.74% |
| 3 | 52.42% | 85.98% | 42.80% | 83.47% |
| **Average** | **44.67%** | **88.82%** | **28.24%** | **88.01%** |
| **SEM** | **10.92%** | **3.73%** | **8.64%** | **3.05%** |
| **P-value** **(two-tailed)** | **0.0187** | | **0.0029** | |

**(B)** **Log-phase cells shifted to old medium for 15 min.**

| **Experiment #** | **Clone 1** | | **Clone 2** | |
| --- | --- | --- | --- | --- |
|  | **Shifted to old medium** | **No medium shift** | **Shifted to old medium** | **No medium shift** |
| 1 | 73.39% | 9.27% | 79.18% | 8.11% |
| 2 | 70.47% | 2.92% | 78.81% | 4.94% |
| 3 | 91.90% | 5.02% | 82.08% | 2.52% |
| **Average** | **78.59%** | **5.74%** | **80.02%** | **5.19%** |
| **SEM** | **6.71%** | **1.87%** | **1.03%** | **1.62%** |
| **P-value**  **(two-tailed)** | **0.0005** | | **<0.0001** | |
